# Pars plana vitrectomy in vitreous hemorrhage with or without Intravitreal Bevacizumab a comparative overview

**DOI:** 10.12669/pjms.341.12683

**Published:** 2018

**Authors:** Syed Muhammad Faisal, Muhammad Ali Tahir, Alyscia Miriam Cheema, Muhammad Ijaz Anjum

**Affiliations:** 1 Syed Muhammad Faisal, MBBS, FCPS (Ophth). Consultant Ophthalmologist, Ali Trust Eye Hospital, Karachi, Pakistan; 2 Muhammad Ali Tahir, MBBS, FCPS (Ophth), FCPS (Vitreoretina). Consultant Retinal Surgeon, Department of Ophthalmology, Jinnah Post Graduate Medical Centre, Karachi, Pakistan; 3 Alyscia Miriam Cheema, MBBS, FCPS, FRCS. Associate Professor and Head, Department Of Ophthalmology, Jinnah Post Graduate Medical Centre, Karachi, Pakistan; 4 Muhammad Ijaz Anjum, MBBS, MCPS, FCPS, FRCS. Chief Consultant, Ali Trust Eye Hospital, Karachi, Pakistan

**Keywords:** Advanced diabetic eye disease, Avastin, Bevacizumab, Vitrectomy

## Abstract

**Objective:**

To compare the success in patients having vitreous hemorrhage undergoing pars plana vitrectomy with or without preoperative intravitreal injection of Bevacizumab.

**Methods:**

This Randomized controlled trial was conducted at Department of Ophthalmology, Jinnah Postgraduate Medical Centre. Karachi. Duration of study was six months from January 2010 to June 2010. In this study 56 patients of advanced diabetic eye disease were divided into two groups. Patients in Group-A underwent three ports pars plana vitrectomy with preoperative intravitreal injection of Bevacizumab (Avastin) 1.25mg/0.05ml, 3.5mm from the limbus seven days before surgery and in Group-B patients underwent vitrectomy without preoperative intravitreal Bevacizumab (Avastin). Intraoperative bleeding was monitored in both groups and was graded as no bleeding, mild bleeding and severe bleeding. The results were statistically analyzed through computer software SPSS 17.

**Results:**

Twenty eight patients in Group-A who were given an injection of intravitreal Bevacizumab (Avastin) before surgery, intraoperative bleeding monitored was, no bleeding in 17 cases (60.7%), mild was observed in 6 cases (21.4%) and severe bleeding requiring diathermy to stop was observed in only 5 cases (17.9%). 28 patients in Group-B that underwent surgery without Avastin no bleeding was observed in only 2 cases (7.1%), mild in 6 cases (21.4%) and severe in 20 cases (71.4%).

**Conclusions:**

Intravitreal injection of Bevacizumab (Avastin) was effective before vitrectomy in the surgical management of Advanced Diabetic Eye disease.

## INTRODUCTION

Diabetic retinopathy is one of the most common causes of visual impairment in 20 – 64 years old individuals.^1^ In Pakistan, the prevalence of Diabetes Mellitus in adult population greater than 25 years of age is about 9%.^2^ The occurrence of diabetic retinopathy in subjects having diabetes mellitus is estimated to be in between 15.3% to 28.9% according to some studies.^3^,^4^ The prevalence of advanced diabetic eye disease among the patients of diabetic retinopathy was 1.74% in a study conducted in Gaddap.^5^ Advanced diabetic eye disease is characterized by dense non clearing vitreous hemorrhage and /or tractional retinal detachment.^6^ Advanced diabetic eye disease is induced by retinal ischemia that leads to retinal neovascularization. It has been shown that VEGF expression is the main factor responsible for pathologic ocular neovascularization. These findings provide the rationale behind the use of anti-VEGF therapy in retinal vascular diseases associated with neovessels formation.^7^ Bevacizumab (Avastin) is a potent anti-VEGF agent. It is recombinant humanized monoclonal antibody directed against all isoforms of VEGF. It has been used effectively in the treatment of neovascular AMD.^8^,^9^ Its antiangiogenic potential is also useful in the treatment of advanced diabetic eye disease.^10^,^11^ Vitreous hemorrhage due to diabetes was first time treated by vitrectomy in 1970.^12^ The use of Intravitreal Avastin few days before vitrectomy may reduce bleeding intraoperatively. In a comparative study the reduction of bleeding was up to 64% in a group of patients who received intravitreal Avastin before vitrectomy as compared to those who did not receive Avastin.^7^ The rational of our study is to provide health care system adequate knowledge about the usefulness of intravitreal injection of Avastin before vitrectomy in order to reduce the potential intraoperative bleeding during vitrectomy surgery for the management of advanced diabetic eye disease. Reduction in bleeding will reduce surgical time and will provide clear visibility during surgery. Clear visibility can prevent iatrogenic retinal tear formation and number of other complications.^7^-^13^

## METHODS

This randomized control trial was conducted at department of Ophthalmology Jinnah postgraduate Medical Centre, Karachi. It was approved by hospital ethical review committee. The sample size included 56 eyes of 56 patients with advanced diabetic eye disease. Patients having either gender, aging between 45-65 years with non-resolving vitreous hemorrhage for three months even after treatment secondary to diabetes were included in the study however those with previous vitrectomy, any major surgery with in past 3 months, uncontrolled hypertension, known coagulation abnormalities & current use of anticoagulant medications were excluded. Patients who met the benchmark for inclusion were selected through diabetic retina clinic, department of Ophthalmology, Jinnah Post Graduate Medical Centre. Informed and written consent for inclusion into study and about the procedure was taken. Data was entered in data sheets. Two groups of patients were made and after all aseptic measures Group-A patients were given intravitreal injection of 1.25 mg in 0.05 ml Avastin (Bevacizumab) 3.5 mm from the limbus seven days before surgery in the operation theatre. After seven days these patients underwent 23 gauge 3 ports pars plana vitrectomy by experienced surgeon using the Dorc Associate vitrectomy machine. Group-B patients underwent 23 gauge 3 ports pars plana vitrectomy without preoperative intravitreal injection of Avastin using Dorc Associate vitrectomy machine. The same experienced ophthalmic surgeon performed all the surgeries. The amount of intraoperative bleeding was monitored by researcher and was graded as no, mild or severe. No bleeding, if no intraoperative bleeding occurred during surgery, mild if bleeding was stopped by increasing the infusion pressure and severe, if endodiathermy was required to stop the bleeding. Comparison was done between Groups A and B regarding the amount of intraoperative bleeding. No to mild bleeding was taken as success. Data was collected in specially designed data sheets. Since the study was conducted by Government run hospital so the funding was borne by government.

Statistical analysis was performed with computer software package SPSS version 17. Clinical characteristics were summarized in terms of frequency / percentage for qualitative variables i.e. gender, type of diabetes, intraoperative bleeding, retinal tear and success (intraoperative no bleeding/mild bleeding) rate, whereas mean ± S.D for quantitative variables i.e. age, duration of disease, surgical time. Statistical comparison in between study groups were performed by using Chi-square/fisher exact test (for qualitative variables) and Students t-test (for quantitative variables). The level of significance was set at 0.05.

## RESULTS

Fifty six patients were inducted into the study, 28 patients in each Groups A and B. Patients in Group-A received intravitreal injection of Avastin (Bevacizumab) seven days before vitrectomy surgery; patients in Group-B underwent vitrectomy surgery without intravitreal injection of Avastin (Bevacizumab). Out of 28 patients in Group-A 15 was males and 13 were females and in Group-B 11 were males and 17 were females. Mean age in Group-A was 58.1 ± 5.04 years and in Group-B 57.2 ± 5.54 years. The mean duration of diabetes was 6.4 years in Group-A and 7.4 years in Group-B. 22 out of 28 were suffering from IDDM and 6 from NIDDM in Group-A, and in Group-B 23 were suffering from IDDM and 5 from NIDDM. Their demographic data can be studied in Table-I. The two groups were well matched for gender, age, type of DM and site of eye. There was no statistically significant difference in duration of disease (p=0.162).

**Table-I:** Baseline characteristics in the study groups.

Characteristics	Group-A	Group-B	P-value

	Preoperative Avastin	Without Preoperative Avastin	
No of patients	28	28	
	*Gender*		
Male	15 (53.6%)	11 (39.3%)	0.284
Female	13 (46.4%)	17 (60.7%)
Age (years) Mean ± S.D	58.1 ± 5.04	57.2 ± 5.54	0.531
	*Type of Diabetes Mellitus*		
IDDM	22 (78.6%)	23 (82.1%)	0.737
NIDDM	6 (21.4%)	5 (17.9%)
	*Site of Eye*		
Right	15 (53.6%)	13 (46.4%)	0.593
Left	13 (46.4%)	15 (53.6%)	
Duration of disease (years) Mean ± S.D	6.4 ± 2.18	7.4 ± 3.02	0.162
No significant difference was observed (p>0.05)			

Comparison of Intraoperative procedure in the study groups is shown in Table-II, Mean surgical time (mean ± S.D) of Group-A (64.1±10.35) were significantly less as compare to Group-B (80.5 ± 10.22) (p=0.001). Iatrogenic tear incidence was 7.1% in Group-A, whereas, in Group-B it was 28.6% (p=0.036). Amount of bleeding was significantly less in Group-A as compare to Group-B. In Group-A no bleeding was observed in 60.7% of patients, mild in 21.4% and severe in 17.9% whereas, in Group-B no bleeding was observed in 7.1%, mild in 21.4% and severe in 71.4% of patients. Success rate was significantly high 82.1% in Group-A (received intravitreal injection of Avastin seven days before vitrectomy surgery) as compare to Group-B (underwent vitrectomy surgery without intravitreal injection of Avastin) 28.6% (p=0.001).

**Table-II:** Comparison of Intraoperative procedure in the study groups.

Characteristics	Group-A	Group-B	P-value

	Preoperative Avastin	Without Preoperative Avastin	
No of patients	28	28
Surgical time (minutes) Mean ± S.D	64.1 ± 10.35 *	80.5 ± 10.22	0.001

* Significantly less as compared to Group-B (p<0.05)

## DISCUSSION

Use of Avastin (Bevacizumab) prior to diabetic vitrectomies in order to decrease intraoperative bleeding through regression of neovascularization has been common. In this study intraoperative Avastin (Bevacizumab) was injected in an effort to quiet down the fibrovascular proliferation before vitrectomy making surgery easier. In this study, there was marked reduction of intraoperative bleeding in Group-A which was reflected in minimal need to use the endodiathermy.

In our study mean age in Group-A was 58.1 years (range 47-65) and in Group-B it was 57.2 years (range 47-65). The maximum numbers of patients were in their fifth decade. However, in a similar study conducted by Rizzo et al, ages of the 22 patients ranged from 24 to 63 mean age 52 years^7^ In another study conducted by El-Batarny, the mean ages of the patients were 46 with a range of 24-65 and 44 with a range of 23-57 in Groups 1 and 2 respectively.^11^ Yeoh et al., in a study reported mean age of 46 years with a range of 28-61 years.^13^ The similar studies on diabetic patients showed slight variation in the age groups affected in different geographical settings. Since our study was done in a government hospital, larger numbers of our patients were from poor financial background.

The mean duration of diabetes in our study was 6.4 years in Group-A and 7.4 years in Group-B. Mean duration in a study conducted by Avery et al., was 15 years^14^ longer the duration of diabetes, the higher the prevalence of diabetic retinopathy and later on advanced diabetic eye disease. In third world countries like Pakistan shorter duration of diabetes is associated with advanced diabetic eye disease, this may be attributed to multiple reasons like poor literacy rate, patients go to the doctors late due to poor financial conditions, poor compliances with medicines and inappropriate dietary habits. All above mentioned factors lead to rapid progression of diabetic retinopathy.

In severe cases of diabetic retinopathy like advanced diabetic eye disease with thick and multiple layers of proliferative tissues, repeated bleeding from multiple sites may make the operation lengthy and tedious. Also extensive cautery may incite postoperative inflammation. Prolonged elevation of intraocular pressure to stop bleeding may cause corneal edema impairing surgical visualization. Therefore, any agent that can induce regression of neovascularization like intravitreal anti VEGF few days prior to surgery may lead to decrease intraoperative bleeding, facilitate vitrectomy surgery and fibrovascular membrane peeling and reduce the time of surgery. Some studies have reported that it also reduces the incidence of post-operative vitreous hemorrhage.^15^-^21^

In our study the intraoperative reduction in bleeding was statistically significant between two groups. In Group-A that received Avastin before vitrectomy no bleeding was observed in 60.7%, mild was observed in 21.4% and severe requiring diathermy to stop bleeding in only 17.9% of cases. In Group-B that underwent surgery without Avastin no bleeding was observed in only 7.1% and severe in 71.4%. In a similar study conducted by Rizzo et al., in a Group that received Avastin no bleeding was observed in 54% of cases, mild in 27% and severe in 18% and in a group that didn't receive Avastin no bleeding was observed in 18% only and severe in 81.8%.^7^ In another study conducted by El-Batarny, 15 patients were placed in each group. In non Avastin group severe bleeding requiring diathermy to stop bleeding was encountered in nearly all 15 cases whereas, in a group with preoperative Avastin no bleeding was seen in 13.3%, mild was encountered in 60% and severe bleeding was observed in only 26.6%.^11^ In a study conducted in India by Nagpal M et al., no bleeding was observed in 56.6% of cases, mild and severe was encountered in 21.8% each in a group of patients with preoperative Avastin whereas, no bleeding was observed in only 18%, mild in 27% and severe requiring diathermy was encountered in almost 55% of cases.^22^

Reduction in surgical time was obvious between two groups. In our study mean surgical time in Group-A with preoperative Avastin was 64 minutesS.D±10.35 and in Group-B that is without preoperative Avastin it was 80.5 minutes S.D±10.22 minutes. In a similar study by Rizzo et al., reduction in surgical time in Avastin Group-was also significant. Mean surgical time in Avastin group was 57 minutes and in non Avastin group it was 83 minutes.^7^ Mean surgical time in another study conducted by El-Batarny in non Avastin group was 93.3 S.D±11.6 minutes and in preoperative Avastin group it was 61.6S.D±14.5 minutes.^11^ Reduction in surgical time is due to lesser bleeding in group with preoperative Avastin. In our study Group-A patient required less number of total diathermy applications and also there was minimal blurring of the surgeon's operating field suggesting facilitation of the surgery, on the other hand, higher range of diathermy applications in Group-B suggests greater intraocular bleeding making the surgery difficult and time consuming. Moreover the shorter duration of surgery decreased the probability of intraoperative damage.

Greater bleeding during surgery obscures the operating field of the surgeon leading to higher incidence of iatrogenic tears in retina of the patients. In our study incidence of iatrogenic tears during surgery in Group-A with preoperative Avastin was 7.1% and in Group-B it was 18.6%. In a study by Rizzo et al, there was on intraoperative retinal tear in a group with Avastin out of 11 cases and in other group without preoperative Avastin incidence of retinal tears were 36.3%.^7^ In a similar study done by El-Batarny intraoperative retinal tears were reported in 40% in Group-1 without Avastin and in 20% in Group-2 with Avastin given preoperatively.^11^

## CONCLUSION

This study provides objective proof that the intravitreal injection of Avastin (bevacizumab) before vitrectomy for vitreous hemorrhage and tractional retinal detachment facilitates the surgery by reducing the surgical time, decrease the intraoperative bleeding and need for hemoststic measures and reduces the incidence of iatrogenic retinal tears. Therefore, we recommend intravitreal injection of Avastin especially before vitrectomies in diabetic patients having vitreous hemorrhage and/or tractional retinal detachment.

### Author`s Contribution

**SMF:** Conceived the study and managed acquisition of data and gave intravitreal injections.

**MAT:** Drafting the article and critical review.

**AC:** Supervised the study and performed surgeries.

**MIA:** Contributed in literature search, analysis and interpretation.
